# Supplementary material for: A lethal ORC ATPase mutation is suppressed by alterations in ORC and RNA Pol II transcription components
Source: PLoS One. 2026 Sep 30;21(9):e0349465. doi: 10.1371/journal.pone.0349465 (PMC13626347; doi:10.1371/journal.pone.0349465)
Supplement: Supplementary file 1 [file pone.0349465.s007.docx]

S1 Table. Yeast strains used in this study.

| Strain | Additional information | Genotype | Source |
| --- | --- | --- | --- |
| yRH101 | Base Strain for ORC protein purifications | *MATa ade2-1 trp1-1 leu2-3,112 his3-11,15 ura3-1 can1-100 BAR1::HisG LYS2::HisG PEP4Δ::KanMX* | Bell Lab |
| yAZ55 | ORC WT protein purification | *yRH101 URA3::pAA01 (GAL1,10- UbiSort-Flag-Orc1, Orc2) HIS3::pLM001 (GAL1,10-Orc3, Orc4) TRP1::pJF18 (GAL1,10-Orc5-E104K, Orc6)* | Amasino et. al. 2023, Zhang et. al. 2023 |
| yLM001 | ORC Orc4 R267A protein purification | *yRH101 URA3::pAA01 (GAL1,10- UbiSort-Flag-Orc1, Orc2) HIS3::pLM001 (GAL1,10-Orc3, Orc4-R267A) TRP1::pJF18 (GAL1,10-Orc5-E104K, Orc6) ORC4::V5-ORC4* | This Study |
| yLM002 | ORC Orc4 R267A with Orc4-*sofr* mutation protein purification | *yRH101 URA3::pAA01 (GAL1,10- UbiSort-Flag-Orc1, Orc2) HIS3::pLM001 (GAL1,10-Orc3, Orc4-R267A-P225S) TRP1::pJF18 (GAL1,10-Orc5-E104K, Orc6) ORC4::V5-ORC4* | This Study |
| yLM003 | ORC Orc4 R267A with Orc1-*sofr* mutation protein purification | *yRH101 URA3::pAA01 (GAL1,10- UbiSort-Flag-Orc1-E495K, Orc2) HIS3::pLM001 (GAL1,10-Orc3, Orc4-R267A) TRP1::pJF18 (GAL1,10-Orc5-E104K, Orc6) ORC4::V5-ORC4* | This Study |
| yLM004 | ORC Orc4 R267A with Orc3-*sofr* mutation protein purification | *yRH101 URA3::pAA01 (GAL1,10- UbiSort-Flag-Orc1, Orc2) HIS3::pLM001 (GAL1,10-Orc3-P481L, Orc4-R267A) TRP1::pJF18 (GAL1,10-Orc5-E104K, Orc6) ORC4::V5-ORC4* | This Study |
| yLM005 | ORC Orc4 R267A with Orc5-*sofr* mutation protein purification | *yRH101 URA3::pAA01 (GAL1,10- UbiSort-Flag-Orc1, Orc2) HIS3::pLM001 (GAL1,10-Orc3, Orc4-R267A) TRP1::pJF18 (GAL1,10-Orc5-E104K, Orc6) ORC4::V5-ORC4* | This Study |
| yLM006 | ORC Orc4 R267A protein with Orc6-*sofr* mutation purification | *yRH101 URA3::pAA01 (GAL1,10- UbiSort-Flag-Orc1-E495K, Orc2) HIS3::pLM001 (GAL1,10-Orc3, Orc4-R267A) TRP1::pJF18 (GAL1,10-Orc5, Orc6-E305K) ORC4::V5-ORC4* | This Study |
| yLM007 | ORC Orc1-K485T protein purification | *yRH101 URA3::pAA01 (GAL1,10- UbiSort-Flag-Orc1-K485T, Orc2) HIS3::pLM001 (GAL1,10-Orc3, Orc4) TRP1::pJF18 (GAL1,10-Orc5,Orc6) ORC4::V5-ORC4* | This Study |
| yLM008 | *ORC4* plasmid-shuffle strain base strain | *MATalpha ade2-1 trp1-1 leu2-3,112 his3-11,15 ura3-1 can1-100 ORC4::TRP1 p(URA3, ORC4,ADE2)* | This Study |
| yLM009 | *WT ORC4* plasmid-shuffle strain | *yLM007 605::V5-ORC4* | This Study |
| yLM010 | *orc4-R267A* plasmid-shuffle strain | *yLM007 605::V5-orc4-R267A* HIS3 | This  Study |
| yLM011 | *WT ORC4* plasmid-shuffle *orc1*-*sofr* strain | *yLM009 orc1-E495K* | This  Study |
| yLM012 | *WT ORC4* plasmid-shuffle *orc3*-*sofr* strain | *yLM009 orc3-P481L* | This  Study |
| yLM013 | *WT ORC4* plasmid-shuffle *orc4*-*sofr* strain | *yLM007 605::V5-orc4-P225S* | This  Study |
| yLM014 | *WT ORC4* plasmid-shuffle *orc5*-*sofr* strain | *yLM009 orc5-E104K* | This  Study |
| yLM015 | *WT ORC4* plasmid-shuffle *orc*6-*sofr* strain | *yLM009 orc6-E304K* | This  Study |
| yLM016 | *WT ORC4* plasmid-shuffle *toa2*-*sofr* strain | *yLM010 toa2-G16E* | This  Study |
| yLM017 | *orc4-R267A* plasmid-shuffle *orc1*-*sofr* strain | *yLM010 orc1-E495K* | This  Study |
| yLM018 | *orc4-R267A* plasmid-shuffle *orc3*-*sofr* strain | *yLM010 orc3-P481L* | This  Study |
| yLM019 | *orc4-R267A* plasmid-shuffle *orc4*-*sofr* strain | *yLM007*  *605::V5-orc4-R267A-P225S* | This  Study |
| yLM020 | *orc4-R267A* plasmid-shuffle *orc5*-*sofr* strain | *yLM010 orc5-E104K* | This  Study |
| yLM021 | *orc4-R267A* plasmid-shuffle *orc6*-*sofr* strain | *yLM010 orc6-E304K* | This  Study |
| yLM022 | *orc4-R267A* plasmid-shuffle *toa2*-*sofr* strain | *yLM010 toa2-G16E* | This  Study |
| AIAy19 | *ORC1* plasmid-shuffle strain base strain | *MATa ade2-1 trp1-1 leu2-3,112 his3-11,15 ura3-1 can1-100 ORC1::hisG p(URA3, ORC1)* | Bell Lab |
| yLM023 | WT-*ORC1* plasmid-shuffle strain | *AIAy19 605::V5-ORC1* | This  Study |
| yLM024 | *orc1-K485T* plasmid-shuffle strain | *AIAy19 605::V5-orc1-K485T* | This  Study |
| yLM025 | *orc1-R616A* plasmid-shuffle strain | *AIAy19 605::V5-orc1-R616A* | This  Study |
| yLM026 | *orc1-K485T* plasmid-shuffle *orc1*-*sofr* strain | *AIAy19*  *605::V5-orc1-K485T-E495K* | This  Study |
| yLM027 | *orc1-K485T* plasmid-shuffle *orc3*-*sofr* strain | *yLM022 orc3-P481L* | This  Study |
| yLM028 | *orc1-K485T* plasmid-shuffle *orc4*-*sofr* strain | *yLM022 orc4-P225S* | This  Study |
| yLM029 | *orc1-K485T* plasmid-shuffle *orc5*-*sofr* strain | *yLM022 orc5-E104K* | This  Study |
| yLM030 | *orc1-K485T* plasmid-shuffle *orc6*-*sofr* strain | *yLM022 orc6-E304K* | This  Study |
| yLM031 | *orc1-K485T* plasmid-shuffle *toa2*-*sofr* strain | *yLM022 toa2-G16E* | This  Study |
| yLM032 | *orc1-R616A* plasmid-shuffle *orc1*-*sofr* strain | *AIAy19*  *605::V5-orc1-R616A-E495K* | This  Study |
| yLM033 | *orc1-R616A* plasmid-shuffle *orc3*-*sofr* strain | *yLM023 orc3-P481L* | This  Study |
| yLM034 | *orc1-R616A* plasmid-shuffle *orc4*-*sofr* strain | *yLM023 orc4-P225S* | This  Study |
| yLM035 | *orc1-R616A* plasmid-shuffle *orc5*-*sofr* strain | *yLM023 orc5-E104K* | This  Study |
| yLM036 | *orc1-R616A* plasmid-shuffle *orc6*-*sofr* strain | *yLM023 orc6-E305K* | This  Study |
| yLM037 | *orc1-R616A* plasmid-shuffle *toa2*-*sofr* strain | *yLM023 toa2-G16E* | This  Study |
| OAy044 | *orc1-161* strain | *MATa trp1-1 leu2-3,112 his3-11,15 ura3-1 can1-100 orc1-161* | Aparicio et. al. 1997 |
| yLM038 | *orc1-161* *ORC1* strain | *OAy044 LEU2::ORC1* | This  Study |
| yLM039 | *orc1-161* *orc1*-*sofr* strain | *OAy044 orc1-E495K* | This  Study |
| yLM040 | *orc1-161* *orc3*-*sofr* strain | *OAy044 orc3-P481L* | This  Study |
| yLM041 | *orc1-161* *orc4*-*sofr* strain | *OAy044 orc4-P225S* | This  Study |
| yLM042 | *orc1-161* *orc5*-*sofr* strain | *OAy044 orc5-E104K* | This  Study |
| yLM043 | *orc1-161* *orc6*-*sofr* strain | *OAy044 orc6-E304K* | This  Study |
| yLM044 | *orc1-161* Toa2-*sofr* strain | *OAy044 toa2-G16E* | This  Study |
| JRY4325 | *orc2-1* strain | *MATa ade2-1 trp1-1 leu2-3,112 his3-11,15 ura3-1 can1-100 orc2-1* | Aparicio et. al. 1997 |
| yLM045 | *orc2-1* *ORC2* strain | *JRY4325 HIS3::ORC2* | This study |
| yLM046 | *orc2-1* *orc1*-*sofr* strain | *JRY4325 orc1-E495K* | This study |
| yLM047 | *orc2-1* *orc3*-*sofr* strain | *JRY4325 orc3-P481L* | This study |
| yLM048 | *orc2-1* *orc4*-*sofr* strain | *JRY4325 orc4-P225S* | This study |
| yLM049 | *orc2-1* *orc5*-*sofr* strain | *JRY4325 orc5-E104K* | This study |
| yLM050 | *orc2-1* *orc6*-*sofr* strain | *JRY4325 orc6-E305K* | This study |
| yLM051 | *orc2-1* *toa2*-*sofr* strain | *JRY4325 toa2-G16E* | This study |
| yLM063 | *orc4-R267A* *toa1-V251F* plasmid-shuffle strain | *yLM010 toa1-V251F* | This study |
| yLM064 | *orc4-R267A* *spt15-K133R* plasmid-shuffle strain | *yLM010 spt15-K133R* | This study |
| yLM354 | *orc4-R267A* *sua7-F289S* plasmid-shuffle strain | yLM010 *sua7-F289S* | This study |
| yLM356 | *orc4-R267A* *rpo21-G1437D* plasmid-shuffle strain | yLM010 *rpo21-G1437D* | This study |
| yLM357 | *orc4-R267A* *ssl2-H508R* plasmid-shuffle strain | yLM010 *ssl2-H508R* | This study |
| yLM359 | *orc4-R267A* *spt15-Y139A* plasmid-shuffle strain | yLM010 *spt15-Y139A* | This study |
| yLM362 | *orc4-R267A* *sua7-K201I* plasmid-shuffle strain | yLM010 *sua7-K201I* | This study |
| yLM365 | *orc4-R267A* *DST1::KAN* plasmid-shuffle strain | yLM010 *DST1::KAN* | This study |
| yLM369 | *orc4-R267A* *ssl2-VIEK* plasmid-shuffle strain | yLM010 *ssl2-VIEK* | This study |
| yLM373 | *orc4-R267A* *spt15-A86D* plasmid-shuffle strain | yLM010 *spt15-A86D* | This study |
| yLM374 | *orc4-R267A* *spt15-R107A* plasmid-shuffle strain | yLM010 *spt15-R107A* | This study |
| yLM377 | *orc4-R267A* *spt15-E188A* plasmid-shuffle strain | yLM010 *spt15-E188A* | This study |
| yLM379 | *orc4-R267A* *rpo21-M818I* plasmid-shuffle strain | yLM010 *rpo21-M818I* | This study |
| yHR050 | *CDC6 plasmid shuffle strain* | *S288C CDC6::KanMX4 pRS416-CDC6-RI* | This study |
| yLM150 | *CDC6 overexpression;*  *CDC6* plasmid shuffle strain | yHR050 2289::*GAL-CDC6* | This study |
| yLM159 | *Cdc6 overexpression;*  *ORC4* plasmid-shuffle strain | *MATa ade2-1 trp1-1 leu2-3,112 his3-11,15 ura3-1 can1-100 ORC4::TRP1 p(URA3, ORC4,ADE2) 605::ORC4* 2289::*GAL-CDC6* | This study |
| yLM160 | Cdc6 overexpression *orc4-R267A* plasmid-shuffle strain | *MATa ade2-1 trp1-1 leu2-3,112 his3-11,15 ura3-1 can1-100 ORC4::TRP1 p(URA3, ORC4,ADE2) 605::orc4-R267A BAR1::NAT* 2289::*GAL-CDC6* | This study |
| yLM162 | *ORC1* plasmid shuffle; Cdc6 Overexpression | AIAy19 *605::ORC1* 2289::GAL-Cdc6 *BAR1::NAT* *Mata* | This study |
| yLM197 | *ORC1* plasmid shuffle; Cdc6 Overexpression | AIAy19 *605::orc1-K485T* *2289::GAL-CDC6* | This study |
| yLM201 | *ORC1* plasmid shuffle; Cdc6 Overexpression | AIAy19 *605::orc1-R616A* *2289::GAL-CDC6* | This study |
