# Supplementary material for: A lethal ORC ATPase mutation is suppressed by alterations in ORC and RNA Pol II transcription components
Source: PLoS One. 2026 Sep 30;21(9):e0349465. doi: 10.1371/journal.pone.0349465 (PMC13626347; doi:10.1371/journal.pone.0349465)
Supplement: Supplementary file 2 [file pone.0349465.s008.docx]

S2 Table. Plasmids used in this study

| Plasmid | Additional Information | Description | Source |
| --- | --- | --- | --- |
| bRA89 | Used for CRISPR-Cas9 editing, express SpCas9-3xNLS, contains SNR52 promoter | *HYGMX, SpCas9-3xNLS, SNR2* promoter | Anand et. al. 2017 |
| p*ARS1*/WT | Used for gel-shift assay, contains A, B1 and B2 elements | Digest with EcoRI and HindIII, PAGE purify, Klenow Labeling | Marahrens and Stillman 1992 |
| p*ARS1*/*858-865* | Used for gel-shift assay, deletion of A element | Digest with EcoRI and HindIII, PAGE purify, Klenow Labeling | Marahrens and Stillman 1992 |
| pUC19-ARS1 | Used as PCR template for reconstituted helicase loading assay |  | Kang et. al. 2015 |
| pUC19-ARS1 (A-/B2-) | Used as PCR template for reconstituted helicase loading assay |  | Kang et. Al. 2015 |
| pLM003 | Protein expression | *HIS3::pJF17 (GAL1,10-Orc3, Orc4-R267A)* | This study |
| pLM004 | Protein expression | *HIS3::pJF17 (GAL1,10-Orc3, Orc4-R267A-P225S)* | This study |
| pLM005 | Protein expression | *HIS3::pJF17 (GAL1,10-Orc3-P481L, Orc4-R267A)* | This study |
| pLM006 | Protein expression | *URA3::pJF19 (GAL1,10-UbiSort-3xFlag-Orc1-E495K, Orc2)* | Amasino et. al. 2023 |
| pLM007 | Protein expression | *TRP1::pJF18 (GAL1,10-Orc5-E104K, Orc6)* | This study |
| pLM008 | Protein expression | *TRP1::pJF18 (GAL1,10-Orc5, Orc6-E305K)* | This study |
