# Supplementary material for: A lethal ORC ATPase mutation is suppressed by alterations in ORC and RNA Pol II transcription components
Source: PLoS One. 2026 Sep 30;21(9):e0349465. doi: 10.1371/journal.pone.0349465 (PMC13626347; doi:10.1371/journal.pone.0349465)
Supplement: Supplementary file 3 [file pone.0349465.s009.docx]

S3 Table. Oligos and gRNAs used in this study

| Plasmid/Oligo/gRNA | Additional Information | Description | Source |
| --- | --- | --- | --- |
| Forward Oligo for 1.3 kbp origin-DNA template | Used in reconstituted helicase-loading assay | 5’GGAAAGCGGGCAGTGAGCGC-3’ | IDT |
| Reverse Oligo for 1.3 kbp origin-DNA template | Used in reconstituted helicase-loading assay | 5’- /5Biosg/GATCGGTGCGGGCCTCTTCGC -3’ | IDT |
| Orc1-gRNA1 | *orc1-E495K*  CRISPR cloning | 5’-AACCGTAAGGGAAGTCGTAA-3’ | This study |
| Orc3-gRNA1 | *orc3-P481L*  CRISPR cloning | 5’-GTAAGAAGGGAAAATCGACT-3’ | This study |
| Orc4-gRNA1 | *orc4-P225S*  CRISPR cloning | 5’-ATACATTTGCTGGGCCTGTG-3’ | This study |
| Orc5-gRNA1 | *orc5-E104K*  CRISPR cloning | 5’-CTGTAAAGGATCGTAATCTG-3’ | This study |
| Orc6-gRNA1 | *orc6-E305K*  CRISPR cloning | 5’-GGCAAAAGCCCTGAGGAAAA-3’ | This study |
| Toa2-gRNA1 | *toa2-G16E*  CRISPR cloning | 5’-TATATCGTAGAAGTACTATA-3’ | This study |
| Toa1-gRNA1 | *toa1-V251F*  CRISPR cloning | 5’- TGATAAAGTCACAAGAACAA-3’ | This study |
| Spt15-gRNA1 | *Spt15-Y139A and K133R* CRISPR cloning | 5’-AAGTGAGGATGACTCAAAGC-3’ | This study |
| Spt15-gRNA2 | *Spt15-E188A* CRISPR cloning | 5’-AGGAAACAATTCTGGCTCAT-3’ | This study |
| Spt15-gRNA3 | *Spt15-A86D and R107A* CRISPR cloning | 5’-GATGACAGCAGCAAAACGCT-3’ | This study |
| Sua7-gRNA1 | *Sua7-K201I and F289S* CRISPR cloning | 5’-GATCGGAAAGGGTGAAACCA-3’ | This study |
| Sua7-gRNA2 | *Sua7-F289S* CRISPR cloning | 5’-CCACTTTCGCTGCGGTAATA-3’ | This study |
| Rpo21-gRNA1 | *Rpo21-M818I* CRISPR cloning | 5’-CTTCACGACCACCCATTGCA-3’ | This study |
| Rpo21-gRNA2 | *Rpo21-G1437D* CRISPR cloning | 5’-GATGATCGATGAGGAGTCAC-3’ | This study |
| Ssl2-gRNA1 | *Ssl2-H508R* CRISPR cloning | 5’-GTTCCAGACAGAATCCGGTT-3’ | This study |
| Ssl2-gRNA2 | *Ssl2-V552I/E556K* CRISPR cloning | 5’-GACACCAAACCTCTGCACAT-3’ | This study |
