# Supplementary material for: A lethal ORC ATPase mutation is suppressed by alterations in ORC and RNA Pol II transcription components
Source: PLoS One. 2026 Sep 30;21(9):e0349465. doi: 10.1371/journal.pone.0349465 (PMC13626347; doi:10.1371/journal.pone.0349465)
Supplement: Supplementary file 4 [file pone.0349465.s010.docx]

**A lethal ORC ATPase mutation is suppressed by alterations in ORC and RNA Pol II transcription components**

**Summary**

This manuscript presents an elegant genetic analysis of the essential in vivo function of ORC ATP hydrolysis through a suppressor screen of the lethal orc4-R267A mutant. The study identifies suppressor mutations in multiple ORC subunits as well as components of the general transcription machinery, revealing an unexpected genetic interaction between ORC ATPase function and transcription. A major strength of the work is the systematic combination of genetics and biochemistry to demonstrate that suppression does not occur through restoration of ATPase activity, ATP-stimulated DNA binding, or helicase loading. These findings provide compelling evidence that the essential function of ORC ATP hydrolysis extends beyond its previously characterized biochemical activities. The suppressor analysis is thorough, the genetic conclusions are well supported, and the identification of transcription-related suppressors is potentially of broad interest to the replication field. The principal weakness of the manuscript is that the mechanistic basis of suppression remains unresolved. Although the authors propose plausible models involving replication-transcription conflicts or transcription-dependent effects on origin licensing, the current data do not distinguish among these possibilities. In addition, the observation of an ATP-dependent DNA-binding defect in ORC4R appears to differ from prior studies of this mutant and would benefit from further discussion. Nevertheless, these limitations do not diminish the validity of the core findings. The data support the major conclusions of the manuscript, and the remaining concerns can be addressed through clarification of interpretation, improved discussion of alternative mechanisms, and reconciliation with previous literature. I therefore recommend publication after minor revision.

**Major Comments**

1) Previous work from Bell and colleagues reported that ORC-4R (orc4-R267A) retains normal origin-DNA binding in vitro and in vivo as assessed by DNase I footprinting and ChIP (Bowers et al., 2004). In contrast, the current study concludes that ORC4R is defective in ATP-stimulated DNA binding. Authors mention this discrepancy. It would benefit the readers if they can discuss this apparent discrepancy and clarify whether differences in assay design, salt conditions, DNA substrates, ATP concentrations, ORC preparations, or quantification methods account for the differing conclusions.
2) The manuscript establishes a genetic interaction between ORC ATPase-site mutants and the general transcription machinery. However, the proposed mechanisms involving replication-transcription conflicts and transcription-dependent disruption of helicase loading remain speculative. The authors should more clearly separate experimentally supported conclusions from mechanistic models and discuss whether existing data favor one model over the other. Can the authors discuss whether the suppressor phenotypes are more consistent with reduced replication-transcription conflicts during S phase or with improved origin licensing during G1, and whether available genetic or biochemical evidence favors one model over the other?

**Minor Comments**

1. The methods should include details on statistics, number of biological replicates and error bars as variability is expected from growth assays to helicase loading assay and EMSA as well. Therefore, by including the statistical details the inferences will be strengthened.
2. The authors conclude that ORC4R exhibits increased sensitivity to elevated salt concentrations, consistent with a DNA-binding defect. However, WT helicase loading also appears reduced at higher salt concentrations. Quantitative comparison of the relative salt sensitivities of WT and mutant complexes would strengthen this conclusion.
3. The DNA-binding assays display multiple shifted DNA-bound species. Please clarify whether all shifted species were included in the quantification and whether the identities of the distinct shifted complexes are known or have been investigated.
4. ORC4R exhibits a measurable defect in ATP-stimulated DNA binding but retains substantial helicase-loading activity. It would be helpful if the authors discussed how the magnitude of the DNA-binding defect relates to the relatively modest reduction in helicase loading and how this compares with previous models of ORC ATPase function.
5. Several suppressor mutations rescue viability despite failing to restore helicase loading and, in some cases, further reducing loading efficiency. This is an important observation. The authors may wish to more explicitly discuss whether the helicase-loading assay strictly measures productive loading or whether altered stability of loading intermediates could potentially contribute to the observed signal.
6. The discussion states that the DNA-binding and helicase-loading defects of ORC4R and ORC1K would translate into fewer replication forks in vivo. While this is a reasonable interpretation, replication origin utilization and fork density were not directly measured. The wording should be softened or explicitly identified as a prediction.
7. Line 161 states that- “the orc1, orc5, orc6 and toa2 alleles, were sensitive to 200 mM hydroxyurea (S1A and S1B Figs). While actually Supp Fig. 1A and 1B along with the figure legend specify 50mM of HU was used. Authors should correct this discrepancy.
8. Lines 369-371-“ The TOA1 allele, toa1-V251F, two TBP alleles (spt15-Y139A and spt15-E188A) and an RNA Pol II allele (rpo21-G1437D) were the strongest suppressors.” But the figure 6 does not have any mutant named “spt15-Y139A” the mutant mentioned is “spt15-Y137A”, this is probably a typographical mistake, authors should correct.
