# Supplementary material for: A lethal ORC ATPase mutation is suppressed by alterations in ORC and RNA Pol II transcription components
Source: PLoS One. 2026 Sep 30;21(9):e0349465. doi: 10.1371/journal.pone.0349465 (PMC13626347; doi:10.1371/journal.pone.0349465)
Supplement: Supplementary file 5 [file pone.0349465.s012.docx]

We thank the reviewers the reviewers for their careful reading of the manuscript and their feedback. We have addressed each comment below. In addition, for Fig. 3C, we performed two additional repeats of the experiments, and quantified and plotted the results.

**A lethal ORC ATPase mutation is suppressed by alterations in ORC and RNA Pol II transcription components**

**Summary**

This manuscript presents an elegant genetic analysis of the essential in vivo function of ORC ATP hydrolysis through a suppressor screen of the lethal orc4-R267A mutant. The study identifies suppressor mutations in multiple ORC subunits as well as components of the general transcription machinery, revealing an unexpected genetic interaction between ORC ATPase function and transcription. A major strength of the work is the systematic combination of genetics and biochemistry to demonstrate that suppression does not occur through restoration of ATPase activity, ATP-stimulated DNA binding, or helicase loading. These findings provide compelling evidence that the essential function of ORC ATP hydrolysis extends beyond its previously characterized biochemical activities. The suppressor analysis is thorough, the genetic conclusions are well supported, and the identification of transcription-related suppressors is potentially of broad interest to the replication field. The principal weakness of the manuscript is that the mechanistic basis of suppression remains unresolved. Although the authors propose plausible models involving replication-transcription conflicts or transcription-dependent effects on origin licensing, the current data do not distinguish among these possibilities. In addition, the observation of an ATP-dependent DNA-binding defect in ORC4R appears to differ from prior studies of this mutant and would benefit from further discussion. Nevertheless, these limitations do not diminish the validity of the core findings. The data support the major conclusions of the manuscript, and the remaining concerns can be addressed through clarification of interpretation, improved discussion of alternative mechanisms, and reconciliation with previous literature. I therefore recommend publication after minor revision.

**Major Comments**

1) Previous work from Bell and colleagues reported that ORC-4R (orc4-R267A) retains normal origin-DNA binding in vitro and in vivo as assessed by DNase I footprinting and ChIP (Bowers et al., 2004). In contrast, the current study concludes that ORC4R is defective in ATP-stimulated DNA binding. Authors mention this discrepancy. It would benefit the readers if they can discuss this apparent discrepancy and clarify whether differences in assay design, salt conditions, DNA substrates, ATP concentrations, ORC preparations, or quantification methods account for the differing conclusions.

We added an additional paragraph to the discussion to clarify the observations of Bowers et al., 2004 and our data focusing on the differences between the experimental conditions.

**Page 19, Line 409 to 420**

“The ORC4R DNA binding defect (Fig 3B) appears at odds with previous studies of this mutation [25]. That study used DNase I protection assays to show that ORC4R protects the same *ARS1* region as wild-type ORC in the presence of ATP. In contrast, using EMSA we observed a clear defect in *ARS1* DNA binding in the presence (but not the absence) of ATP. The most likely explanation of the difference is that the EMSA assays used much higher salt (50mM vs 150 mM KCl) and competitor DNA (0.08 vs 0.3 mg/ml) concentrations. In addition, the DNAse I protection and EMSA assays measure DNA binding very differently (e.g. one is a solution assay, and the other is an in-gel assay), and it appears that the EMSA assay is more sensitive to DNA binding defects. In this regard, it is noteworthy that ORC was originally detected by DNAse I protection assays [3] whereas the detection of ORC-DNA complex in the EMSA assay requires modified assay conditions [51,75,76].”

2) The manuscript establishes a genetic interaction between ORC ATPase-site mutants and the general transcription machinery. However, the proposed mechanisms involving replication-transcription conflicts and transcription-dependent disruption of helicase loading remain speculative. The authors should more clearly separate experimentally supported conclusions from mechanistic models and discuss whether existing data favor one model over the other. Can the authors discuss whether the suppressor phenotypes are more consistent with reduced replication-transcription conflicts during S phase or with improved origin licensing during G1, and whether available genetic or biochemical evidence favors one model over the other?

We believe that our data does not allow us to draw specific conclusions regarding the mechanism of replication-transcription conflicts. We have added that the *toa1-V251F* allele is also a suppressor of NC2, an essential complex that represses non-specific or pervasive transcription. However, modulation of NC2 could impact either of the mechanisms that we propose to explain the suppressive effect of the transcriptional mutants. There is no data connecting the rest of the transcription alleles tested for *orc4-R267A* suppression to pervasive transcription or transcription-replication conflicts.

Accordingly, we have added the following two sentences to the last paragraph of the discussion section. Page 22, Lines 480 to 483

“Interestingly, the *toa1-V251F* allele is a suppressor of Negative Cofactor 2 complex (NC2) mutations, an essential complex that inhibits PIC assembly to prevent pervasive transcription [59,86–89]. This function, however, could impact either or both mechanisms described above.”

**Minor Comments**

1. The methods should include details on statistics, number of biological replicates and error bars as variability is expected from growth assays to helicase loading assay and EMSA as well. Therefore, by including the statistical details the inferences will be strengthened.

We have added additional details to the methods section.

Methods Section, Pooled-linkage analysis and whole-genome sequencing, Page 24, last sentence of second paragraph
“All python scripts and bam files can be found in a Zenodo repository (DOI: 10.5281/zenodo.19625098).”

Methods section, Plasmid-shuffle assay, Page 25, last two sentences

“Ten-fold serial dilutions were plated and incubated for 2-5 days on the indicated media at the indicated temperatures. Two independent clones of each strain were grown and tested. Only one representative clone is shown.”

Methods section, Electrophoretic Mobility Shift Assay, Page 26, last three sentences

“All DNA templates are WT *ARS1* and all reactions used ATP unless indicated otherwise. All images were collected on a GE Amersham Typhoon Biomolecular Imager. A second, more slowly migrating shifted species was occasionally observed and is consistent with two ORC molecules bound to a single DNA, as reported previously [3,10]. Quantification reflects the lower (single-ORC) shifted band.”

Methods section, ATPase assay, Page 26, second sentence minor correction

“A 90 min-timepoint sample without ORC was used to subtract the background signal from all samples.”

Methods Section, Image quantification, Page X, reworked paragraph

“ImageJ (v1.54g) was used to quantify images from electrophoretic-mobility shift, ATPase, and helicase-loading assays. For each lane, an intensity profile was generated using the Gel Analysis tool, a straight-line baseline was subtracted, and band signal was measured as the area under each peak. ATPase reactions were quantified as the fraction of ADP relative to total nucleotide (ADP / [ADP + ATP]) and corrected against a no-ORC background control (Fig 3A). Gel shift reactions were quantified as bound fraction (top band/ [top band + bottom band]) and normalized to the WT+ATP sample within each gel (Fig 3B); when two shifted bands were resolved, only the lower band was quantified. Helicase-loading reactions (Fig 3C, SFig 4, SFig 5) were quantified by integrating the total intensity of the Mcm2-7 subunit region within each lane and corrected against a no-ORC background control. R (v4.4.3) was used for downstream analysis and plotting.”

Methods Section, Data analysis and visualization, Page 27, new section

“R 4.4.3 was used to align and plot the multiple sequence alignments in S2 and S3 Figs and to generate the plots in Figs 3A, S4A, and S4B. Plots in Fig 3A, Fig 3B and Fig 3C, SFig 4B display the mean and standard deviation, as error bars, of three replicate experiments. The ggplot2 R package was used to generate all the plots. All R scripts can be found in a Zenodo repository (DOI: 10.5281/zenodo.19625098).”

2. The authors conclude that ORC4R exhibits increased sensitivity to elevated salt concentrations, consistent with a DNA-binding defect. However, WT helicase loading also appears reduced at higher salt concentrations. Quantitative comparison of the relative salt sensitivities of WT and mutant complexes would strengthen this conclusion.

We thank the reviewer for this point. In response to the reviewer’s point, we quantitatively analyzed the same data plotted in SFig. 4B and found that the ORC4R and ORC WT helicase loading are equally salt sensitive. We have revised the text to reflect this analysis. We originally tested different salt concentrations to assess the effect of the *orc4-sofa* allele on ORC4R loading. The conclusion that two of the suppressor alleles improve ORC4R loading remains unaffected.

We have modified a sentence in the Results section, subsection “The *sofa* mutations have different effects on the biochemical activities of ORC4R protein”, Page 12, Line 247

“Under higher-salt conditions that showed the greatest ORC4R loading defect, the ORC4R^Orc1-^*^sofa^* and ORC4R^Orc4-^*^sofa^* proteins show improved loading but neither reached WT levels (Fig 3C).”

And Page 13, Line 282-283

“The ORC4R protein has a consistent loading deficit relative to WT across all salt concentrations (S5D, S4A and S4B Figs).”

3. The DNA-binding assays display multiple shifted DNA-bound species. Please clarify whether all shifted species were included in the quantification and whether the identities of the distinct shifted complexes are known or have been investigated.

We have added clarifications to the methods section to say that we only quantified the faster migrating band. Although our experiments do not specifically address whether the species is a two-ORC bound DNA molecule, this is almost certainly the case, as has been reported previously (references 3 and 10).

4. ORC4R exhibits a measurable defect in ATP-stimulated DNA binding but retains substantial helicase-loading activity. It would be helpful if the authors discussed how the magnitude of the DNA-binding defect relates to the relatively modest reduction in helicase loading and how this compares with previous models of ORC ATPase function.

We have added two sentences in the second paragraph of the Discussion section. Page 19, lines 401 to 405

“The ORC4R DNA-binding defect may be masked in the reconstituted loading assay where the high local DNA concentration on the bead surface could compensate for mutant complexes with weakened DNA-binding activity. This bead-specific effect could explain why the DNA-binding defect appears less pronounced in the loading assay than in the in-gel-based EMSA experiments.”

5. Several suppressor mutations rescue viability despite failing to restore helicase loading and, in some cases, further reducing loading efficiency. This is an important observation. The authors may wish to more explicitly discuss whether the helicase-loading assay strictly measures productive loading or whether altered stability of loading intermediates could potentially contribute to the observed signal.

Our current set of experiments do not address whether the loading products are active in steps downstream of helicase loading but other studies, including our own, have shown that products from the reconstituted helicase loading assay are competent for activation and unwinding (references 23 and 54). Because some of the suppressors restore viability without restoring helicase loading activity *in vitro*, we hypothesize that some *in vivo* factors are not recapitulated in the *in vitro* loading assay. We think our discussion touches on potential factors (for example, the chromatin environment) without over speculation.

We added a sentence to the second paragraph of the discussion section.

Page 19, Line 405-408

“Because our experiments do not address whether the ORC4R-loaded complexes are functional for downstream helicase activation and initial DNA unwinding events, it remains possible that the suppressors improve the functionality of the ORC4R-loaded helicases.”

6. The discussion states that the DNA-binding and helicase-loading defects of ORC4R and ORC1K would translate into fewer replication forks in vivo. While this is a reasonable interpretation, replication origin utilization and fork density were not directly measured. The wording should be softened or explicitly identified as a prediction.

We agree with the reviewers and have adjusted the sentence to read as a prediction.

Eighth paragraph of the discussion section, Page 22, Line 470-472

“The ORC4R and ORC1K proteins show DNA-binding and helicase loading defects, which would be predicted to generate less replication forks *in vivo.*”

7. Line 161 states that- “the orc1, orc5, orc6 and toa2 alleles, were sensitive to 200 mM hydroxyurea (S1A and S1B Figs). While actually Supp Fig. 1A and 1B along with the figure legend specify 50mM of HU was used. Authors should correct this discrepancy.

We adjusted the value to 50 mM hydroxyurea. The legend and the figures remain unchanged.

8. Lines 369-371 - “The TOA1 allele, toa1-V251F, two TBP alleles (spt15-Y139A and spt15-E188A) and an RNA Pol II allele (rpo21-G1437D) were the strongest suppressors.” But the figure 6 does not have any mutant named “spt15-Y139A” the mutant mentioned is “spt15-Y137A”, this is probably a typographical mistake, authors should correct.

We adjusted the figure 6 label to spt15-Y139A. The text remains the same.
