# Supplementary material for: A lethal ORC ATPase mutation is suppressed by alterations in ORC and RNA Pol II transcription components
Source: PLoS One. 2026 Sep 30;21(9):e0349465. doi: 10.1371/journal.pone.0349465 (PMC13626347; doi:10.1371/journal.pone.0349465)
Supplement: Peer review history

# Peer review: Decision Letter 0

**DOI:** 10.1371/journal.pone.0349465.r001

**Authors:** Sue Cotterill

15 Jun 2026

Dear Dr. Bell,

Please submit your revised manuscript by Jul 30 2026 11:59PM. If you will need more time than this to complete your revisions, please reply to this message or contact the journal office at plosone@plos.org. When you're ready to submit your revision, log on to https://www.editorialmanager.com/pone/ and select the 'Submissions Needing Revision' folder to locate your manuscript file.

- A letter that responds to each point raised by the academic editor and reviewer(s). You should upload this letter as a separate file labeled 'Response to Reviewers'.
- A marked-up copy of your manuscript that highlights changes made to the original version. You should upload this as a separate file labeled 'Revised Manuscript with Track Changes'.
- An unmarked version of your revised paper without tracked changes. You should upload this as a separate file labeled 'Manuscript'.

If you would like to make changes to your financial disclosure, please include your updated statement in your cover letter. Guidelines for resubmitting your figure files are available below the reviewer comments at the end of this letter.

If applicable, we recommend that you deposit your laboratory protocols in protocols.io to enhance the reproducibility of your results. Protocols.io assigns your protocol its own identifier (DOI) so that it can be cited independently in the future. For instructions see: https://journals.plos.org/plosone/s/submission-guidelines#loc-laboratory-protocols. Additionally, PLOS ONE offers an option for publishing peer-reviewed Lab Protocol articles, which describe protocols hosted on protocols.io. Read more information on sharing protocols at https://plos.org/protocols?utm_medium=editorial-email&utm_source=authorletters&utm_campaign=protocols.

As the corresponding author, your ORCID iD is verified in the submission system and will appear in the published article. PLOS supports the use of ORCID, and we encourage all coauthors to register for an ORCID iD and use it as well. Please encourage your coauthors to verify their ORCID iD within the submission system before final acceptance, as unverified ORCID iDs will not appear in the published article. *Only*  the individual author can complete the verification step; PLOS staff *cannot*  verify ORCID iDs on behalf of authors.

We look forward to receiving your revised manuscript.

Kind regards,

Sue Cotterill

Academic Editor

PLOS One

Journal Requirements:

When submitting your revision, we need you to address these additional requirements.

1.Please ensure that your manuscript meets PLOS ONE's style requirements, including those for file naming. The PLOS ONE style templates can be found at

https://journals.plos.org/plosone/s/file?id=wjVg/PLOSOne_formatting_sample_main_body.pdf and

https://journals.plos.org/plosone/s/file?id=ba62/PLOSOne_formatting_sample_title_authors_affiliations.pdf

2. Please note that PLOS One has specific guidelines on code sharing for submissions in which author-generated code underpins the findings in the manuscript. In these cases, we expect all author-generated code to be made available without restrictions upon publication of the work. Please review our guidelines at https://journals.plos.org/plosone/s/materials-and-software-sharing#loc-sharing-code and ensure that your code is shared in a way that follows best practice and facilitates reproducibility and reuse.

3. Thank you for stating the following financial disclosure:

“S.P.B. is an investigator with the Howard Hughes Medical Institute.”

Please state what role the funders took in the study.  If the funders had no role, please state: "The funders had no role in study design, data collection and analysis, decision to publish, or preparation of the manuscript."

If this statement is not correct you must amend it as needed.

Please include this amended Role of Funder statement in your cover letter; we will change the online submission form on your behalf.

4. Please note that your Data Availability Statement is currently missing the repository name and/or the DOI/accession number of each dataset OR a direct link to access each database. If your manuscript is accepted for publication, you will be asked to provide these details on a very short timeline. We therefore suggest that you provide this information now, though we will not hold up the peer review process if you are unable.

5. Please amend either the abstract on the online submission form (via Edit Submission) or the abstract in the manuscript so that they are identical.

6. PLOS ONE now requires that authors provide the original uncropped and unadjusted images underlying all blot or gel results reported in a submission’s figures or Supporting Information files. This policy and the journal’s other requirements for blot/gel reporting and figure preparation are described in detail at https://journals.plos.org/plosone/s/figures#loc-blot-and-gel-reporting-requirements and https://journals.plos.org/plosone/s/figures#loc-preparing-figures-from-image-files. When you submit your revised manuscript, please ensure that your figures adhere fully to these guidelines and provide the original underlying images for all blot or gel data reported in your submission. See the following link for instructions on providing the original image data: https://journals.plos.org/plosone/s/figures#loc-original-images-for-blots-and-gels.

In your cover letter, please note whether your blot/gel image data are in Supporting Information or posted at a public data repository, provide the repository URL if relevant, and provide specific details as to which raw blot/gel images, if any, are not available. Email us at plosone@plos.org if you have any questions.

7. If the reviewer comments include a recommendation to cite specific previously published works, please review and evaluate these publications to determine whether they are relevant and should be cited. There is no requirement to cite these works unless the editor has indicated otherwise.

8. Please review your reference list to ensure that it is complete and correct. If you have cited papers that have been retracted, please include the rationale for doing so in the manuscript text, or remove these references and replace them with relevant current references. Any changes to the reference list should be mentioned in the rebuttal letter that accompanies your revised manuscript. If you need to cite a retracted article, indicate the article’s retracted status in the References list and also include a citation and full reference for the retraction notice.

[Note: HTML markup is below. Please do not edit.]

Reviewers' comments:

Reviewer's Responses to Questions

**Comments to the Author**

1. Is the manuscript technically sound, and do the data support the conclusions?

Reviewer #1: Yes

Reviewer #2: Yes

**********

2. Has the statistical analysis been performed appropriately and rigorously?

Reviewer #1: I Don't Know

Reviewer #2: Yes

**********

3. Have the authors made all data underlying the findings in their manuscript fully available?

The PLOS Data policy

Reviewer #1: Yes

Reviewer #2: Yes

**********

4. Is the manuscript presented in an intelligible fashion and written in standard English?

Reviewer #1: Yes

Reviewer #2: Yes

**********

Reviewer #1: Neither the methods section or figure legends have information on number of biological replicates or statistics or error bars. I have mentioned this as one of my comments for the authors. This is the reason why I "do not know" if the statistical analysis been performed appropriately and rigorously.

Reviewer #2: This is a careful study, analysing suppressors of the orc4-R267A mutation which disrupts ORC ATP hydrolysis. Most of the suppressor mutations affect ORC subunits and some increase helicase loading activity. Surprisingly, none of the suppressors restore ORC ATPase activity. One weak suppressor affects Toa2 which is a component of TFIIA. The mechanism of suppression here is not clarified but may be due to a general reduction in Pol II transcription which in some way compensates for defective ORC function.

Although this study does not really clarify the role of ORC ATPase activity it is suitable for publication PlosOne as it stands.

**********

what does this mean?). If published, this will include your full peer review and any attached files.

If you choose “no”, your identity will remain anonymous but your review may still be made public.

**Do you want your identity to be public for this peer review?**  For information about this choice, including consent withdrawal, please see our Privacy Policy

Reviewer #1: **Yes:**  Shalu Sharma

Reviewer #2: No

**********

[NOTE: If reviewer comments were submitted as an attachment file, they will be attached to this email and accessible via the submission site. Please log into your account, locate the manuscript record, and check for the action link "View Attachments". If this link does not appear, there are no attachment files.]

To ensure your figures meet our technical requirements, please review our figure guidelines: https://journals.plos.org/plosone/s/figures

You may also use PLOS’s free figure tool, NAAS, to help you prepare publication quality figures: https://journals.plos.org/plosone/s/figures#loc-tools-for-figure-preparation.

NAAS will assess whether your figures meet our technical requirements by comparing each figure against our figure specifications.

Attachment Submitted filename: Orc ATPase suppressor screen-PONE_review.docx

# Author comment: Author response to Decision Letter 1

**DOI:** 10.1371/journal.pone.0349465.r002

7 Sep 2026

We thank the reviewers the reviewers for their careful reading of the manuscript and their feedback. We have addressed each comment below. In addition, for Fig. 3C, we performed two additional repeats of the experiments, and quantified and plotted the results.

A lethal ORC ATPase mutation is suppressed by alterations in ORC and RNA Pol II transcription components

Summary

This manuscript presents an elegant genetic analysis of the essential in vivo function of ORC ATP hydrolysis through a suppressor screen of the lethal orc4-R267A mutant. The study identifies suppressor mutations in multiple ORC subunits as well as components of the general transcription machinery, revealing an unexpected genetic interaction between ORC ATPase function and transcription. A major strength of the work is the systematic combination of genetics and biochemistry to demonstrate that suppression does not occur through restoration of ATPase activity, ATP-stimulated DNA binding, or helicase loading. These findings provide compelling evidence that the essential function of ORC ATP hydrolysis extends beyond its previously characterized biochemical activities. The suppressor analysis is thorough, the genetic conclusions are well supported, and the identification of transcription-related suppressors is potentially of broad interest to the replication field. The principal weakness of the manuscript is that the mechanistic basis of suppression remains unresolved. Although the authors propose plausible models involving replication-transcription conflicts or transcription-dependent effects on origin licensing, the current data do not distinguish among these possibilities. In addition, the observation of an ATP-dependent DNA-binding defect in ORC4R appears to differ from prior studies of this mutant and would benefit from further discussion. Nevertheless, these limitations do not diminish the validity of the core findings. The data support the major conclusions of the manuscript, and the remaining concerns can be addressed through clarification of interpretation, improved discussion of alternative mechanisms, and reconciliation with previous literature. I therefore recommend publication after minor revision.

Major Comments

1) Previous work from Bell and colleagues reported that ORC-4R (orc4-R267A) retains normal origin-DNA binding in vitro and in vivo as assessed by DNase I footprinting and ChIP (Bowers et al., 2004). In contrast, the current study concludes that ORC4R is defective in ATP-stimulated DNA binding. Authors mention this discrepancy. It would benefit the readers if they can discuss this apparent discrepancy and clarify whether differences in assay design, salt conditions, DNA substrates, ATP concentrations, ORC preparations, or quantification methods account for the differing conclusions.

We added an additional paragraph to the discussion to clarify the observations of Bowers et al., 2004 and our data focusing on the differences between the experimental conditions.

Page 19, Line 409 to 420

“The ORC4R DNA binding defect (Fig 3B) appears at odds with previous studies of this mutation [25]. That study used DNase I protection assays to show that ORC4R protects the same ARS1 region as wild-type ORC in the presence of ATP. In contrast, using EMSA we observed a clear defect in ARS1 DNA binding in the presence (but not the absence) of ATP. The most likely explanation of the difference is that the EMSA assays used much higher salt (50mM vs 150 mM KCl) and competitor DNA (0.08 vs 0.3 mg/ml) concentrations. In addition, the DNAse I protection and EMSA assays measure DNA binding very differently (e.g. one is a solution assay, and the other is an in-gel assay), and it appears that the EMSA assay is more sensitive to DNA binding defects. In this regard, it is noteworthy that ORC was originally detected by DNAse I protection assays [3] whereas the detection of ORC-DNA complex in the EMSA assay requires modified assay conditions [51,75,76].”

2) The manuscript establishes a genetic interaction between ORC ATPase-site mutants and the general transcription machinery. However, the proposed mechanisms involving replication-transcription conflicts and transcription-dependent disruption of helicase loading remain speculative. The authors should more clearly separate experimentally supported conclusions from mechanistic models and discuss whether existing data favor one model over the other. Can the authors discuss whether the suppressor phenotypes are more consistent with reduced replication-transcription conflicts during S phase or with improved origin licensing during G1, and whether available genetic or biochemical evidence favors one model over the other?

We believe that our data does not allow us to draw specific conclusions regarding the mechanism of replication-transcription conflicts. We have added that the toa1-V251F allele is also a suppressor of NC2, an essential complex that represses non-specific or pervasive transcription. However, modulation of NC2 could impact either of the mechanisms that we propose to explain the suppressive effect of the transcriptional mutants. There is no data connecting the rest of the transcription alleles tested for orc4-R267A suppression to pervasive transcription or transcription-replication conflicts.

Accordingly, we have added the following two sentences to the last paragraph of the discussion section. Page 22, Lines 480 to 483

“Interestingly, the toa1-V251F allele is a suppressor of Negative Cofactor 2 complex (NC2) mutations, an essential complex that inhibits PIC assembly to prevent pervasive transcription [59,86–89]. This function, however, could impact either or both mechanisms described above.”

Minor Comments

1. The methods should include details on statistics, number of biological replicates and error bars as variability is expected from growth assays to helicase loading assay and EMSA as well. Therefore, by including the statistical details the inferences will be strengthened.

We have added additional details to the methods section.

Methods Section, Pooled-linkage analysis and whole-genome sequencing, Page 24, last sentence of second paragraph

“All python scripts and bam files can be found in a Zenodo repository (DOI: 10.5281/zenodo.19625098).”

Methods section, Plasmid-shuffle assay, Page 25, last two sentences

“Ten-fold serial dilutions were plated and incubated for 2-5 days on the indicated media at the indicated temperatures. Two independent clones of each strain were grown and tested. Only one representative clone is shown.”

Methods section, Electrophoretic Mobility Shift Assay, Page 26, last three sentences

“All DNA templates are WT ARS1 and all reactions used ATP unless indicated otherwise. All images were collected on a GE Amersham Typhoon Biomolecular Imager. A second, more slowly migrating shifted species was occasionally observed and is consistent with two ORC molecules bound to a single DNA, as reported previously [3,10]. Quantification reflects the lower (single-ORC) shifted band.”

Methods section, ATPase assay, Page 26, second sentence minor correction

“A 90 min-timepoint sample without ORC was used to subtract the background signal from all samples.”

Methods Section, Image quantification, Page X, reworked paragraph

“ImageJ (v1.54g) was used to quantify images from electrophoretic-mobility shift, ATPase, and helicase-loading assays. For each lane, an intensity profile was generated using the Gel Analysis tool, a straight-line baseline was subtracted, and band signal was measured as the area under each peak. ATPase reactions were quantified as the fraction of ADP relative to total nucleotide (ADP / [ADP + ATP]) and corrected against a no-ORC background control (Fig 3A). Gel shift reactions were quantified as bound fraction (top band/ [top band + bottom band]) and normalized to the WT+ATP sample within each gel (Fig 3B); when two shifted bands were resolved, only the lower band was quantified. Helicase-loading reactions (Fig 3C, SFig 4, SFig 5) were quantified by integrating the total intensity of the Mcm2-7 subunit region within each lane and corrected against a no-ORC background control. R (v4.4.3) was used for downstream analysis and plotting.”

Methods Section, Data analysis and visualization, Page 27, new section

“R 4.4.3 was used to align and plot the multiple sequence alignments in S2 and S3 Figs and to generate the plots in Figs 3A, S4A, and S4B. Plots in Fig 3A, Fig 3B and Fig 3C, SFig 4B display the mean and standard deviation, as error bars, of three replicate experiments. The ggplot2 R package was used to generate all the plots. All R scripts can be found in a Zenodo repository (DOI: 10.5281/zenodo.19625098).”

2. The authors conclude that ORC4R exhibits increased sensitivity to elevated salt concentrations, consistent with a DNA-binding defect. However, WT helicase loading also appears reduced at higher salt concentrations. Quantitative comparison of the relative salt sensitivities of WT and mutant complexes would strengthen this conclusion.

We thank the reviewer for this point. In response to the reviewer’s point, we quantitatively analyzed the same data plotted in SFig. 4B and found that the ORC4R and ORC WT helicase loading are equally salt sensitive. We have revised the text to reflect this analysis. We originally tested different salt concentrations to assess the effect of the orc4-sofa allele on ORC4R loading. The conclusion that two of the suppressor alleles improve ORC4R loading remains unaffected.

We have modified a sentence in the Results section, subsection “The sofa mutations have different effects on the biochemical activities of ORC4R protein”, Page 12, Line 247

“Under higher-salt conditions that showed the greatest ORC4R loading defect, the ORC4ROrc1-sofa and ORC4ROrc4-sofa proteins show improved loading but neither reached WT levels (Fig 3C).”

And Page 13, Line 282-283

“The ORC4R protein has a consistent loading deficit relative to WT across all salt concentrations (S5D, S4A and S4B Figs).”

3. The DNA-binding assays display multiple shifted DNA-bound species. Please clarify whether all shifted species were included in the quantification and whether the identities of the distinct shifted complexes are known or have been investigated.

We have added clarifications to the methods section to say that we only quantified the faster migrating band. Although our experiments do not specifically address whether the species is a two-ORC bound DNA molecule, this is almost certainly the case, as has been reported previously (references 3 and 10).

4. ORC4R exhibits a measurable defect in ATP-stimulated DNA binding but retains substantial helicase-loading activity. It would be helpful if the authors discussed how the magnitude of the DNA-binding defect relates to the relatively modest reduction in helicase loading and how this compares with previous models of ORC ATPase function.

We have added two sentences in the second paragraph of the Discussion section. Page 19, lines 401 to 405

“The ORC4R DNA-binding defect may be masked in the reconstituted loading assay where the high local DNA concentration on the bead surface could compensate for mutant complexes with weakened DNA-binding activity. This bead-specific effect could explain why the DNA-binding defect appears less pronounced in the loading assay than in the in-gel-based EMSA experiments.”

5. Several suppressor mutations rescue viability despite failing to restore helicase loading and, in some cases, further reducing loading efficiency. This is an important observation. The authors may wish to more explicitly discuss whether the helicase-loading assay strictly measures productive loading or whether altered stability of loading intermediates could potentially contribute to the observed signal.

Our current set of experiments do not address whether the loading products are active in steps downstream of helicase loading but other studies, including our own, have shown that products from the reconstituted helicase loading assay are competent for activation and unwinding (references 23 and 54). Because some of the suppressors restore viability without restoring helicase loading activity in vitro, we hypothesize that some in vivo factors are not recapitulated in the in vitro loading assay. We think our discussion touches on potential factors (for example, the chromatin environment) without over speculation.

We added a sentence to the second paragraph of the discussion section.

Page 19, Line 405-408

“Because our experiments do not address whether the ORC4R-loaded complexes are functional for downstream helicase activation and initial DNA unwinding events, it remains possible that the suppressors improve the functionality of the ORC4R-loaded helicases.”

6. The discussion states that the DNA-binding and helicase-loading defects of ORC4R and ORC1K would translate into fewer replication forks in vivo. While this is a reasonable interpretation, replication origin utilization and fork density were not directly measured. The wording should be softened or explicitly identified as a prediction.

We agree with the reviewers and have adjusted the sentence to read as a prediction.

Eighth paragraph of the discussion section, Page 22, Line 470-472

“The ORC4R and ORC1K proteins show DNA-binding and helicase loading defects, which would be predicted to generate less replication forks in vivo.”

7. Line 161 states that- “the orc1, orc5, orc6 and toa2 alleles, were sensitive to 200 mM hydroxyurea (S1A and S1B Figs). While actually Supp Fig. 1A and 1B along with the figure legend specify 50mM of HU was used. Authors should correct this discrepancy.

We adjusted the value to 50 mM hydroxyurea. The legend and the figures remain unchanged.

8. Lines 369-371 - “The TOA1 allele, toa1-V251F, two TBP alleles (spt15-Y139A and spt15-E188A) and an RNA Pol II allele (rpo21-G1437D) were the strongest suppressors.” But the figure 6 does not have any mutant named “spt15-Y139A” the mutant mentioned is “spt15-Y137A”, this is probably a typographical mistake, authors should correct.

We adjusted the figure 6 label to spt15-Y139A. The text remains the same.

Attachment Submitted filename: Response to reviewers.docx

# Editor report: Decision Letter 1

**DOI:** 10.1371/journal.pone.0349465.r003

**Authors:** Sue Cotterill

13 Sep 2026

A lethal ORC ATPase mutation is suppressed by alterations in ORC and RNA Pol II transcription components

PONE-D-26-21033R1

Dear Dr. Bell,

We’re pleased to inform you that your manuscript has been judged scientifically suitable for publication and will be formally accepted for publication once it meets all outstanding technical requirements.

Within one week, you’ll receive an e-mail detailing the required amendments. When these have been addressed, you’ll receive a formal acceptance letter and your manuscript will be scheduled for publication.

An invoice will be generated when your article is formally accepted. Please note, if your institution has a publishing partnership with PLOS and your article meets the relevant criteria, all or part of your publication costs will be covered. Please make sure your user information is up-to-date by logging into Editorial Manager at Editorial Manager® and clicking the ‘Update My Information' link at the top of the page. For questions related to billing, please contact billing support.

If your institution or institutions have a press office, please notify them about your upcoming paper to help maximize its impact. If they’ll be preparing press materials, please inform our press team as soon as possible -- no later than 48 hours after receiving the formal acceptance. Your manuscript will remain under strict press embargo until 2 pm Eastern Time on the date of publication. For more information, please contact onepress@plos.org.

Kind regards,

Sue Cotterill

Academic Editor

PLOS One

Additional Editor Comments (optional):

Reviewers' comments:

# Editor report: Acceptance letter

**DOI:** 10.1371/journal.pone.0349465.r004

**Authors:** Sue Cotterill

PONE-D-26-21033R1

PLOS One

Dear Dr. Bell,

I'm pleased to inform you that your manuscript has been deemed suitable for publication in PLOS One. Congratulations! Your manuscript is now being handed over to our production team.

At this stage, our production department will prepare your paper for publication. This includes ensuring the following:

* All references, tables, and figures are properly cited

* All relevant supporting information is included in the manuscript submission,

* There are no issues that prevent the paper from being properly typeset

You will receive further instructions from the production team, including instructions on how to review your proof when it is ready. Please keep in mind that we are working through a large volume of accepted articles, so please give us a few days to review your paper and let you know the next and final steps.

Lastly, if your institution or institutions have a press office, please let them know about your upcoming paper now to help maximize its impact. If they'll be preparing press materials, please inform our press team within the next 48 hours. Your manuscript will remain under strict press embargo until 2 pm Eastern Time on the date of publication. For more information, please contact onepress@plos.org.

You will receive an invoice from PLOS for your publication fee after your manuscript has reached the completed accept phase. If you receive an email requesting payment before acceptance or for any other service, this may be a phishing scheme. Learn how to identify phishing emails and protect your accounts at https://explore.plos.org/phishing.

If we can help with anything else, please email us at customercare@plos.org.

Thank you for submitting your work to PLOS One and supporting open access.

Kind regards,

PLOS One Editorial Office Staff

on behalf of

Dr Sue Cotterill

Academic Editor

PLOS One
